# A Disaggregation Methodology to Estimate Intake of Added Sugars and Free Sugars: An Illustration from the UK National Diet and Nutrition Survey

**DOI:** 10.3390/nu10091177

**Published:** 2018-08-28

**Authors:** Birdem Amoutzopoulos, Toni Steer, Caireen Roberts, Darren Cole, David Collins, Dove Yu, Tabitha Hawes, Suzanna Abraham, Sonja Nicholson, Ruby Baker, Polly Page

**Affiliations:** 1 MRC Elsie Widdowson Laboratory, Cambridge CB1 9NL, UK; toni.steer@mrc-ewl.cam.ac.uk (T.S.); caireen.roberts@mrc-ewl.cam.ac.uk (C.R.); darren.cole@mrc-ewl.cam.ac.uk (D.Cole); david.collins@mrc-ewl.cam.ac.uk (D.Coll.); doveyu30@hotmail.com (D.Y.); tabby-simone@hotmail.com (T.H.); suzanna.abraham@mrc-ewl.cam.ac.uk (S.A.); sonja.nicholson@mrc-ewl.cam.ac.uk (S.N.); rubybaker@live.co.uk (R.B.); polly.page@mrc-ewl.cam.ac.uk (P.P.); 2 NatCen Social Research, London EC1V 0AX, UK

**Keywords:** sugars, added sugars, free sugars, nutrition survey, dietary recommendations, recipe analysis, ingredients, food composition, method

## Abstract

Various and inconsistent definitions for free and added sugars are used in the consideration and assessment of dietary intakes across public health, presenting challenges for nutritional surveillance, research, and policy. Furthermore, analytical methods to identify those sugars which are not naturally incorporated into the cellular structure of foods are lacking, thus free and added sugars are difficult to estimate in an efficient and accurate way. We aimed to establish a feasible and accurate method that can be applied flexibly to different definitions. Based on recipe disaggregation, our method involved five steps and showed good repeatability and validity. The resulting Free Sugars Database provided data for seven components of sugars; (1) table sugar; (2) other sugars; (3) honey; (4) fruit juice; (5) fruit puree; (6) dried fruit; and (7) stewed fruit, for ~9000 foods. Our approach facilitates a standardized and efficient assessment of added and free sugars, offering benefit and potential for nutrition research and surveillance, and for the food industry, for example to support sugar reduction and reformulation agendas.

## 1. Introduction

Across the world, countries have become progressively more concerned about high intakes of sugars and the link to a number of serious health implications, in particular, the risk of obesity and related diseases (for example CVD, cancer, and type 2 diabetes), incidence of dental caries, and micronutrient dilution associated with the displacement of nutrient-dense foods from the diet [1,2,3,4,5,6,7]. Therefore, increasingly, countries are taking steps to monitor and decrease population sugar intake through public health dietary guidance, targets and recommendations [8], and measures like taxes on sugar-sweetened beverages (UK [9] and Mexico [10]) and sugar reduction and reformulation agendas (UK [11] and Norway [12]). In 2015, the World Health Organization (WHO) issued dietary guidelines with revised recommendations for ‘free sugars’ intake [3]. In the same year the United Kingdom stipulated that no more than 5% of daily energy intake should be derived from ‘free sugars’ [13], following advice from the UK Scientific Advisory Committee on Nutrition (SACN) [2].

Internationally, government and public health policy varies in the types of sugars specified in the detail of dietary guidelines [14]; similarly there are a range of definitions of sugars used in research to identify and assess sugar intake:

- ‘Total sugars’ are defined as the total amount of sugars (mono- and disaccharides) from all food sources, without distinguishing between free and non-free sugars. The sugar declared on nutritional panels of food labels always refers to ‘total sugars’ [15].
- ‘Added sugars’ refer to sucrose, fructose, glucose, starch hydrolysates (glucose syrup, high-fructose syrup) and other isolated sugar preparations used as such, or added during food preparation and manufacturing, according to the European Food Safety Authority (EFSA) [16], and does not capture the sugars present in unsweetened fruit juice or honey [2].
- ‘Free sugars’, a term introduced by WHO, refer to all sugars that are added during food manufacturing and preparation as well as sugars that are naturally present in honey, syrups, fruit juices, and fruit concentrates [3]. In a variant to this, the UK has adapted the ‘free sugars’ definition to exclude the ‘lactose’ in milk and the sugars within the cellular structure of foods (particularly fruits and vegetables) [2], whereas this distinction is not specified by WHO [3].
- ‘Non-milk extrinsic sugars’ (NMES) is a term that has been used for some decades in the UK and includes sugars not contained within the cellular walls of plants, all sugars added to foods, and 50% of the sugars in canned, stewed, dried, or preserved fruits [17].

The estimation of added sugar or free sugar intake is a challenge for researchers. Due to the lack of any simple biomarker [18] or analytical method to objectively measure added or free sugars, data rely on subjective estimations. An added complexity is that it is not possible to distinguish naturally occurring sugars from added sugars from an analytical standpoint [19,20]. There are various indirect methods for estimating the added sugar and free sugar content of food and drinks [19,20,21,22,23,24], however, all these methods have limitations, mainly due to the level of subjectivity and general assumptions involved in decision making (for example all savory breads are sugar-free). In addition, these methods are mostly specific to a single definition of sugars, and thus may not be able to adapt readily to accommodate any changes in the definition. For example, a systematic approach for added sugar estimation described by Louie et al. [19] was used by other researchers for the estimation of free sugars [22]. However modifications were necessary to categorize foods such as fruit juices and canned fruits according to the free sugars definition. Any updates to the free sugars dataset created by Kibblewhite et al. [22], for example introducing a new assumption that fruit purees include free sugars, would not be without a considerable effort in terms of cost and time to remodel the data. As the scientific understanding of sugars evolves, sugar definitions and recommendations are becoming more specific and complex. The food environment is continually evolving, and food manufacturers are reformulating their products in response to consumer demand, government policy, and legislation, including the use of novel sugar replacements and/or multiple sugar ingredients. It is therefore a key challenge for researchers, policy makers, and the food industry to assess, monitor, and evaluate sugar intakes in a standardized and consistent way, and to efficiently and effectively accommodate new definitions and changing dietary recommendations.

To our knowledge, methods allowing a flexible estimation of the added and free sugar content of foods in a rapid and robust way are lacking. Our aim, therefore, was to develop a thorough and efficient method, capable of flexible estimation of added and free sugar content of foods, adaptable to different sugar definitions, responsive to changes in sugar definitions, and which would facilitate regular database updates and provision of food-specific data. The validity and inter-rater repeatability of this method was tested and applied for the purposes of reporting free sugar intake in the UK National Diet and Nutrition Survey (NDNS) Rolling Programme (RP).

## 2. Materials and Methods

The principle of the approach is described in Section 2.1, and details of how this was operationalized specifically for the UK NDNS RP is described in Section 2.2.

### 2.1. Principles of Approach

In order to estimate the intake, consumption or content in a flexible way in accordance with different definitions for sugars not naturally available in foods (see Introduction: paragraph 3, page 2), knowledge of the ingredients that make up a food and the proportion of the ingredients and information on the total sugar content for all ingredients is needed. The total sugars for each ingredient then need to be assigned to sub-categories, for example, sugar from honey, sugar from table sugar, or sugar from other sources. Our method allows for estimation of seven sugar components: (1) sugar in table sugar (sugar-table); (2) sugar in other sugar-based sweeteners such as fructose, glucose syrup, malt extract, golden syrup, maple syrup (sugar-other); (3) sugar in honey (sugar-honey); (4) sugar in fruit and vegetable juice (sugar-fruit juice); (5) sugar in fruit puree (sugar-fruit puree); (6) sugar in stewed fruit (sugar-stewed fruit); and (7) sugar in dried fruit (sugar-dried fruit). Summation of the sugar components are then carried out according to the different sugar definitions e.g., added sugar and free sugar content of individual foods.

**Figure 1:**
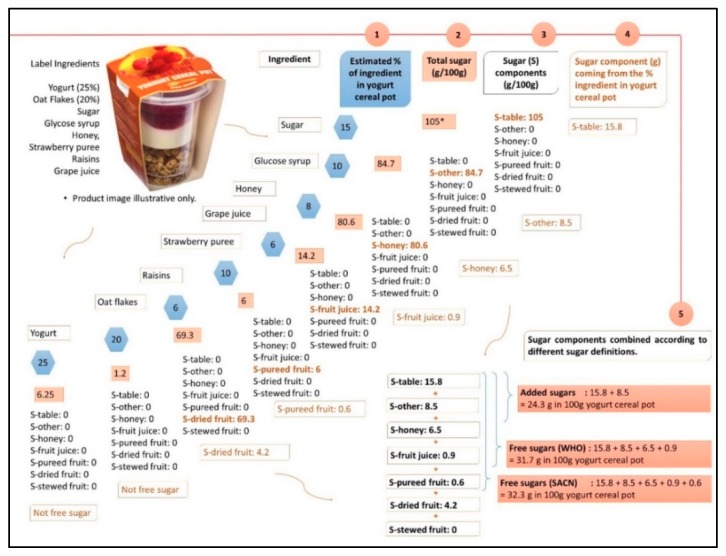
Steps for the estimation of free sugars and added sugars. * Sugar values expressed as monosaccharide equivalents which can exceed 100 g per 100 g of food because on hydrolysis 100 g of a disaccharide such as sucrose gives 105 g monosaccharide (glucose and fructose) [25]. S-table: sugar-table, S-other: sugar-other, S-honey: sugar-honey, S-fruit juice: sugar-fruit juice, S-fruit puree: sugar-fruit puree, S-stewed fruit: sugar-stewed fruit and S-dried fruit: sugar-dried fruit. WHO: World Health Organization. SACN: Scientific Advisory Committee on Nutrition.

Our method involved five steps which are demonstrated below through a fictional example of a yogurt cereal pot (Figure 1).

Step 1. Ingredients and their proportion in the food are identified from ingredient and nutrition information on the food label.

For example, the ingredients and their proportion in the yogurt cereal pot were estimated as; yogurt (25%), oat flakes (20%), sugar (15%), glucose syrup (10%), honey (8%), strawberry puree (10%), raisins (6%), and grape juice (6%).

Step 2. A total sugar value is assigned to each ingredient per 100 g. This can be obtained from food composition tables and databases.

For example, 14.2 g is assigned as the total sugar content of 100 g of grape juice using data from a food composition table (i.e., McCance and Widdowson’s the Composition of Foods [25]).

Step 3. 100% of total sugars of each ingredient is assigned to one of the ‘sugar components’.

For example 100% of total sugars in 100 g of grape juice, 14.2 g, is assigned to ‘sugar-fruit juice’.

Step 4. The proportion of each ingredient making the food is used to calculate a value (g) for each ‘sugar component’ in food using the following formula.

(1) $$g\;\mathrm{sugar}\;\mathrm{component}\;\mathrm{in}\;100\;g\;\mathrm{food}=\;\overset{n}{\underset{i=1}{\sum }}\%\;\mathrm{ingredient}\times g\;\mathrm{sugar}\;\mathrm{component}\;\mathrm{in}\;100\;g\;\mathrm{ingredient}$$

For example, the proportions of grape juice (6%) in a yogurt cereal pot and 14.2 g of total sugar in 100 g grape juice are used to calculate ‘sugar-fruit juice’, which is 0.9 g in 100 g of yogurt cereal pot.

The formula is based on gram (g) ‘sugar component’ values assigned to 100 g of each single ingredient code generated at step 3 where the ‘sugar component’ (g) per 100 g recipe (g/100 g) was calculated by summing the amount (g) of the ‘sugar component’ coming from the proportion of each ingredient in the recipe.

Step 5. The g of ‘sugar components’ in 100 g of foods are then summed in combinations, to give an estimated g ‘added sugars’ and ‘free sugars’ content of 100 g of foods. For example:

Added sugars (EFSA): ‘Sugar-table’ + ‘Sugar-other’
Free sugars (WHO): ‘Sugar-table’ + ‘Sugar-other’ + ‘Sugar-honey’ + ‘Sugar-fruit juice’
Free sugars (SACN): ‘Sugar-table’ + ‘Sugar-other’ + ‘Sugar-honey’ + ‘Sugar-fruit juice’ + ‘Sugar-pureed fruit’
NMES: ‘Sugar-table’ + ‘Sugar-other’ + ‘Sugar-honey’ + ‘Sugar-fruit juice’ + 50% of ‘Sugar-stewed fruit’ + 50% of ‘Sugar-dried fruit’

### 2.2. Methodology for Estimating Sugar Components of Foods: Approach for the UK NDNS RP

As stated in Section 2.1, our method requires knowledge of the ingredients that make up a food, the proportion of the ingredients and information on the total sugar content for all ingredients. To estimate sugar components of foods for the NDNS RP, we used two databases to obtain the ingredients and the sugar values for all foods: the UK NDNS Nutrient Databank [26] and the FSA Recipes Database [27] (see Section 2.3).

Firstly, the Free Sugars Database was created from the version of the FSA Recipes Database hosted at MRC Elsie Widdowson Laboratory, Cambridge, UK. The Recipes Database provides information on the amount of ingredients (ingredient proportion) for each recipe which is either homemade or manufactured food with more than one ingredient. Additional recipes were created in the Recipes Database for foods that had been consumed from 2012 onwards in the NDNS RP through a standard and systematic approach (step 1 of the method outlined in Section 2.1). Users can access the Recipes Database and a more detailed recipes creation procedure from the UK Data Archive [27]. The Recipes Database contained total sugar values for each food (step 2 of the method outlined in Section 2.1). New ingredient codes such as fruit puree were created to support the flexibility required for sugars definitions. The existing recipes in the Recipes Database were reviewed and modified according to food processing relevant to defining sugar intakes, for example, a ‘raw mango’ code in a ‘smoothie’ recipe was replaced by a ‘mango-puree’ code. Additionally, fields for the seven sugar components (described at 2.1. Principles of Approach: paragraph 1, page 3) were created for each ingredient code in the new Free Sugars Database. As not all codes in the database contain sugars, rather than identifying and individually assigning zero values to the ‘sugar components’ for those foods with no sugars and assigning sugar values to sugar-rich foods, zero values were assigned to the ‘sugar components’ of all foods in the Free Sugars Database. Then 100% of ‘total sugars’ were assigned to one of the ‘sugar components’ for the common sugar-rich single ingredient codes (step 3 of the method outlined in Section 2.1). For example, 100% of ‘total sugars’ in ‘apple puree’ was assigned to ‘sugar-fruit puree’. This way the zero values assigned to sugar-rich commodities were overwritten and efforts were focused just on the sugar-rich ingredients. This meant dealing with fewer foods and made the data compilation process more efficient in this large food composition database. However the complexity of this step was when a fruit was processed with multiple methods, for example it is first stewed and then pureed. In this case, the processing method that gave the highest ‘free sugars’ content took precedence over the other methods. For example, ‘total sugars’ in stewed fruit which has been pureed was assigned to ‘sugar-fruit puree’ instead of ‘sugar-stewed fruit’. This approach was more closely aligned with the SACN ‘free sugars’ definition. Although minimal, for a small number of foods, this may compromise the accuracy of the estimates for ‘sugar-stewed fruit’ and ‘sugar-dried fruit’ as it may exclude sugars found in pureed foods. Depending on the primary focus of their study, users can use a separate sugar component like ‘sugar-fruit puree and stewed’ and ‘sugar-fruit puree and dried’ to overcome this limitation.

The sugar-rich single ingredient codes in all foods were manually checked to ensure that a value (not null) was assigned to the ‘sugar component’ (for example ‘raisins’ in a muesli code should have a value assigned for ‘sugar-dried fruit’).

In our Free Sugars Database, an automated background calculation was run to generate a value (g) for each ‘sugar component’ (for example ’sugar-table’) per 100 g of all foods by using the proportion of each ingredient making the food (step 4 of the method outlined in Section 2.1).

For the purpose of estimating free sugars in the NDNS RP, the values of the ‘sugar components’ estimated for foods in the Free Sugars Database were then assigned to the corresponding foods in the UK NDNS Nutrient Databank through a proportioning approach. This step was conducted because of the differences between nutrient values of foods in the Free Sugars Database and the Nutrient Databank because the Nutrient Databank is being updated on an annual basis. For example, in the Free Sugars Database if a yogurt cereal pot has a total sugar value of 40 g/100 g and ‘sugar—table’ value of 15.8 g/100 g, 39.5% of the total sugar is ‘sugar-table’. If the same code in the Nutrient Databank has 35 g/100 g of total sugar, then 39.5% of this is assigned as ‘sugar-table’ which is 13.8 g in 100 g of yogurt cereal pot.

The g of ‘sugar components’ in 100 g of foods were then summed in combinations, to give an estimated g ‘added sugars’ and ‘free sugars’ content per 100 g of foods in the Nutrient Databank (step 5 of the method outlined in Section 2.1).

**Figure 2:**
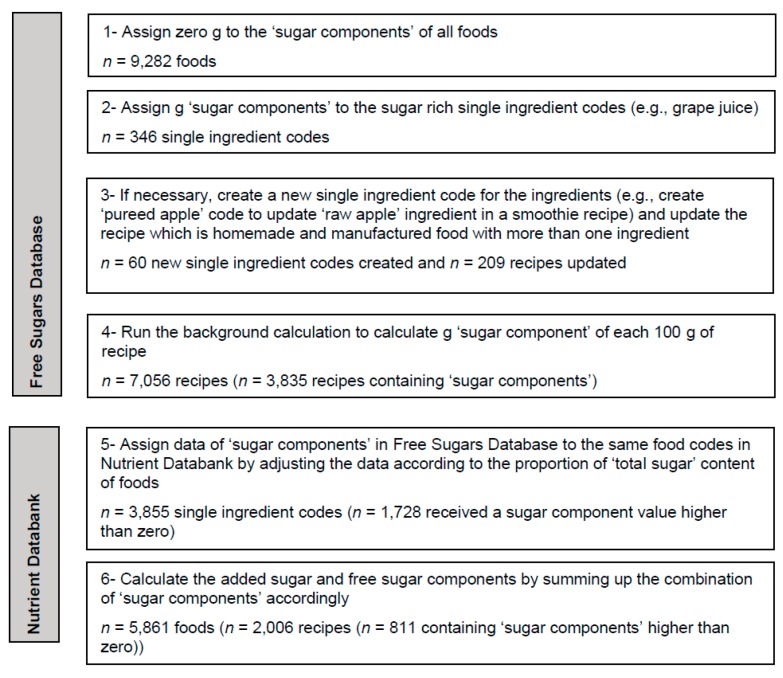
The steps for the estimations of added sugars and free sugars content of foods in the Nutrient Databank for reporting free sugars intake in the United Kingdom National Diet and Nutrition Survey (UK NDNS).

An overview of the methodology for estimating added sugar and free sugar content of foods for the NDNS RP is summarized in Figure 2.

### 2.3. Additional Information on the UK NDNS Nutrient Databank and FSA Recipes Database

#### 2.3.1. UK NDNS Nutrient Databank

The UK NDNS Nutrient Databank [26] holds the food composition data which are used to estimate food consumption and nutrient intake in the NDNS RP. The Nutrient Databank includes approximately 5861 food codes (March 2018). All food codes contain nutrient composition but only a small proportion of food codes contain the individual ingredients which make up the food (called recipes). Some of the codes are based on average nutrient values influenced by market leader brands (for example breakfast biscuits, any brand) but in some cases they are based on a single brand (for example Kellogg’s breakfast biscuit). The nutrient values are populated based on the data gathered from Public Health England’s programme of nutrient analysis (chemical and indirect analysis) of foods, and, for the purposes of the NDNS RP, they are regularly updated and extended based on manufacturers’ data, gathered through food labels to address missing data. The food composition data is managed by a data management system, Nutrient Databank v1.32.0. (©VS Atkins 2002, Minneapolis, MN, USA), which was initially developed by ATKINS for the Foods Standards Agency, and is maintained by the contracted NDNS RP consortium (specifically the MRC Elsie Widdowson Laboratory (Cambridge, UK)) in conjunction with Public Health England. The system has a number of features such as background calculations which enable automated updates of nutrient composition of recipes from single ingredient commodities. For example if a nutrient value of cream changes, this will update all recipe codes containing cream.

#### 2.3.2. Recipes Database

The Recipes Database, which can be accessed online through the UK data archive [27], consists of approximately 8397 foods of which 6960 are recipes reported in UK national nutrition surveys from 1992 including the NDNS RP 2008–2012. As described above, the Recipes Database provides information on the amount of ingredients (ingredient proportion) for each homemade and manufactured food with more than one ingredient, whereas a food in the UK Nutrient Databank is most likely to be a basic food which lacks detail about ingredient information. For example, a cornflakes code in the Nutrient Databank may provide only nutrient composition information (for example energy, protein), whereas, in addition, the Recipes Database provides information on the proportion of the constituent ingredients (for example % of sugar, % of corn flour). In this way, the Recipes Database provides necessary ingredient details for the step required to determine the quantity of sugars in the foods in the Nutrient Databank. The version of Recipe Database hosted in MRC Elsie Widdowson Laboratory includes food substances such as nutrient supplements in addition to the foods available in the online version of Recipes Database available in the UK data archive [27]. It consists of 9282 foods of which 7056 are recipes and it is fully compatible with Nutrient Databank as both databases are managed by the same data management system (Nutrient Databank v1.32.0) and both use the same food name and food identification code for all foods and the same food composition data for ingredients.

### 2.4. Inter-Rater Repeatability

To prove the rigour of our method we tested whether two independent data assistants in our team could estimate the same added sugar and free sugar values for the same foods. As the first step, 50 food products were identified which reflected items available in the UK marketplace and contained at least one of the added sugar and free sugar components (total sugar value split into categories, for example, sugar from honey, sugar from table sugar). Out of these 50 products, 41 were foods including yogurts with fruits, cereals, pies, cereal bars, dairy desserts, sweet spreads, sauces, canned fruits, instant foods, biscuits, puddings and sweets and 9 were drinks including fruit juices, fruit drinks, soft drinks, energy drinks, squash and instant drinks. The data assistants independently created recipes and generated added sugar and free sugar data for the 50 products by applying the method described in Section 2.2 and using the ingredient and nutrition information on food labels to describe and update the values according to the same protocol. The sugar content of individual products estimated by the two compilers were compared using paired sample *t*-tests.

### 2.5. Validity

By measuring the validity, we wanted to test the closeness of the estimated values to the known values created in the inter-rater repeatability exercise. The known values refer to the amount of sugar components (g/100 g) in the product formulations (*n* = 50). The validation test was conducted independently from the inter-repeatability exercise, however the recipes created by one of the data analysts in the inter-rater repeatability test were used as the validation batch in the validation exercise. This was a feasible way to create formulations of which the proportion of ingredients (%) and the amount of sugar components (g/100 g) is known. Using these recipes, a researcher created the label information for the 50 formulations (validation batch) which would give the ingredient and nutrient profile information in the same way that they would be displayed on a usual product label. The label information of the validation batch were then provided to a data analyst in the team (independent from the first data analyst and the researcher) who was asked to generate recipes and to estimate added sugar and free sugar data by applying the method described in Section 2.2. The estimated values (g/100 g) were then compared to the known values of formulations (g/100 g) in order to test the validity of method by another researcher. A Bland-Altman plot was also created to assess the level of agreement between the two sets of values. Paired *t*-tests were used to assess the difference between known and estimated data to determine the accuracy of the method.

## 3. Results

### 3.1. Estimation of Added Sugar and Free Sugar Content of Foods Based on Disaggregation Methodology

The version of the Recipe Database hosted in MRC Elsie Widdowson Laboratory consists of 9282 foods of which 7056 (76%) are recipes. The Free Sugars Database, devised for this study in 2018, included 9342 foods (76% are recipes) of which 4241 (45%) contained a value of sugar components higher than zero. Overall, values for sugar components, including zero values, were assigned to 5861 foods (34% of which were recipes) in the Nutrient Databank (described in Section 2.3.1) of which 2539 (43%) contained a value of sugar components higher than zero (Figure 2).

#### 3.1.1. Inter-Rater Repeatability

Overall, there was no statistically significant difference between values estimated by two data analysists for all sugar components across 50 products (*p* = 0.12–0.80) (Table 1). For added sugars and free sugars the difference between values was less than 0.15 g. Among all individual sugar components, the largest difference was for sugar-other (difference of means −0.21, *p* = 0.36) which was expected as there is no straightforward way to distinguish other sugars from table sugars using the information on food labels. Out of 50 products, 35 (70%) had a less than ± 1 g difference between the added sugar and free sugar values estimated by the two data analysts. The main differences between compilers arose from the variance in understanding of the product properties—such as the use of weight loss factors, selection of sugar-rich ingredients, and the type of ingredients as applicable to sugar definitions (for example malt extract is considered to contain added sugars in our study) or the characteristics of food processing (for example selection of boiled, roasted or stewed fruit code). The main differences were seen in two breakfast cereals with fruits and fruit snack rolls and this was probably due to the extensive list of the ingredients including similar multiple sugar-rich ingredients.

#### 3.1.2. Validity

**Figure 3:**
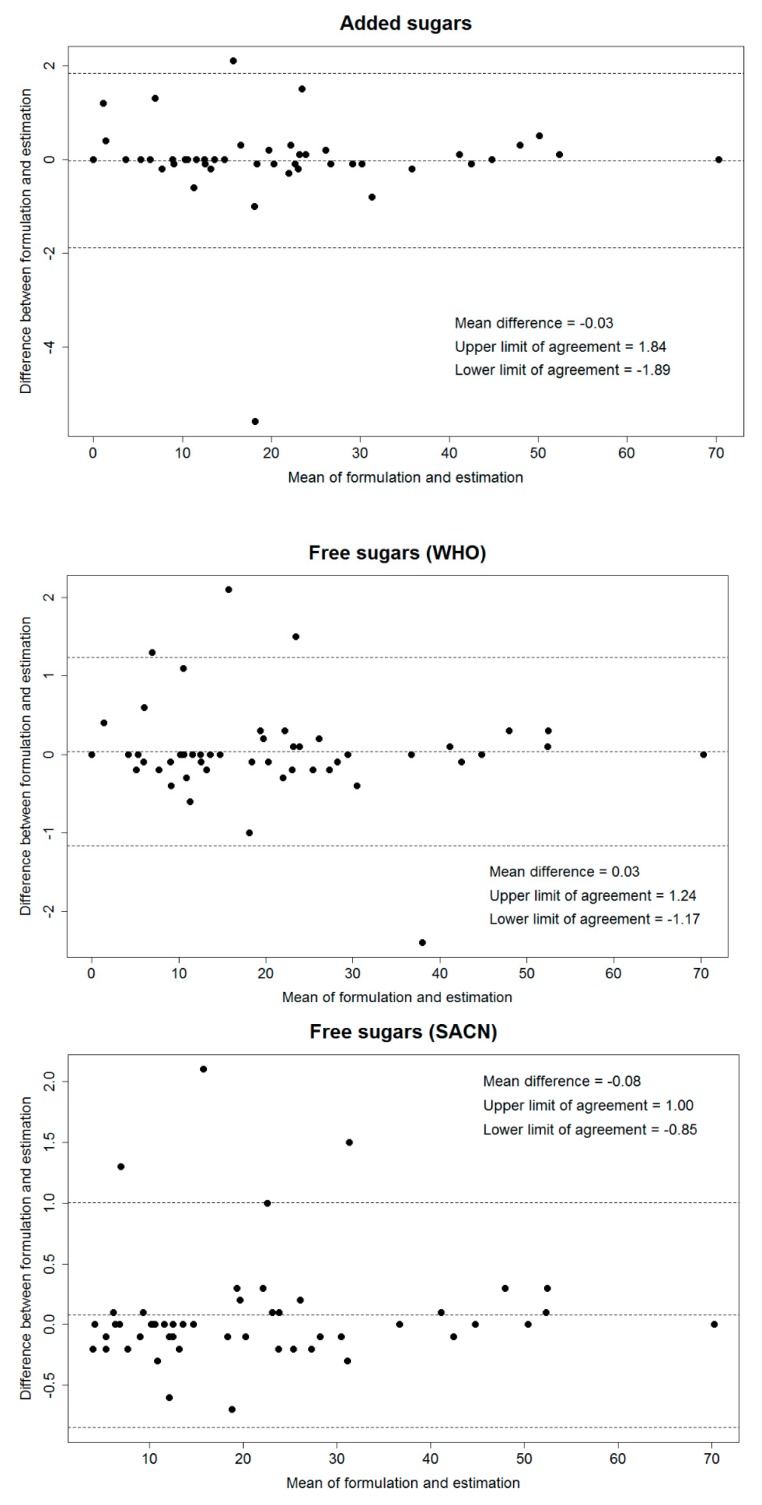
Bland and Altman plot for difference in added sugars and free sugars values (g/100 g) between known formulations and estimation of a data analyst for 50 food items. Added sugars: sugar-table + sugar-other; Free sugars (WHO): Added sugars + sugar-honey + sugar-fruit juice; Free sugars (SACN): Free sugars (WHO) + sugar-pureed fruit. WHO: World Health Organization. SACN: Scientific Advisory Committee on Nutrition.

Results from the validation test on 50 product formulations are shown in Table 2. There was no statistically significant difference between the values of known formulation and the values estimated by a data analyst for all sugar components, indicating that there was excellent agreement between these values. Although the largest differences occurred with table sugar, other sugar, honey, and stewed fruit, the magnitude of the difference was small (mean difference ranged from −0.16 to 0.14 g), and even smaller for the added sugar and free sugar combinations which are added sugars, free sugars (WHO) and free sugars (SACN) (mean difference ranged from −0.03 to 0.08). Not surprisingly, the largest difference among the added sugar and free sugar combinations was for free sugars (SACN) (mean difference 0.08 g) as the definition is more expanded (Table 2). The results of Bland and Altman assessment for added sugars, free sugars (WHO), and free sugars (SACN) are shown in Figure 3. For added sugars and free sugars, only 2–4 foods (4–8%) among the 50 formulations had a difference between the known formulation values and estimated values outside the limit of agreement, and the highest difference was in a fruit snack roll (difference −2.4 g/100 g) and a cereal bar with apricot (difference 2.1 g/100 g) which contained multiple sugar-rich ingredients (Not shown in Table 2).

## 4. Discussion

### 4.1. Estimating the Added Sugar and Free Sugar Content of Foods

Our method presents an accurate, efficient and flexible way to estimate the added sugar and free sugar content of foods. We used a recipe approach to streamline and refine the complexity of the estimation process which should minimize the subjectivity and improve the overall accuracy of the method especially for complex product formulations. Further disaggregation of sugar values to sugar components (sugar-table, sugar-other, sugar-honey, sugar-fruit juice, sugar-fruit puree, sugar-stewed fruit, and sugar-dried fruit) was a new approach to estimate the added sugar and free sugar content of foods which is adaptable to different sugar definitions as well as responding to food specific research questions such as sugar derived from dried fruits. Once this type of analysis is created, it is likely to add to the longevity of the food composition database because it can be flexible to the changing definition of sugars.

Our method demonstrates a more efficient and sensitive estimation of added sugars and free sugars compared to previous studies [19,21,28]. Generalized assumptions are minimized and the disaggregation approach is applied to all foods in the database. This is important as comparison studies [19,21] have indicated significant differences in the estimation of added sugar intake with methods relying on default values.

In total, our method involved only five steps and assigned sugar component values, higher than zero, to just 406 single ingredient codes (4.3% of foods) in order to estimate the sugar component values for 9282 foods. This is a substantially simplified and more efficient approach compared to the greater number of steps (10 to 59 steps) used and less variety of sugar components generated in previous methods [19,20,22,28].

### 4.2. Repeatability and Validity

A strength of our method is its good repeatability and relative validity demonstrating its suitability for use as a standard method to estimate added sugar and free sugar content of food items. Our reliability study covered 50 foods all of which were based on complex product formulations including the combination of free and non-free sugars derived from various ingredients and this indicates high repeatability of our method even for complex foods. Therefore, we would be confident that one person could undertake the estimations. The issues which cause differences between data analysts, for example difference in understanding of the food properties and processing, and extensive list of multiple sugar-rich ingredients, can be resolved easily by development of procedures to guide and quality assure decision making, and a querying system where compilers can flag and resolve ambiguities via discussion. It would also be good practice if the recipe disaggregation of complex foods is quality checked by a second complier, at least for a random sample of commonly consumed foods.

To our knowledge, only one previous study has tested the validity of their method. This was when estimating free sugars in 20 beverages [23] and the method performed less well for products containing multiple sugar-rich ingredients, similar to our method. However, further instructions on choosing the most suitable ingredient from the database may help to alleviate this limitation. Users may also need to consider the effect on accuracy when deciding which definition to use. Higher levels of validity were attained for simpler definitions: the order of the accuracy of estimations from high to low in the validation study were; added sugars > free sugars (WHO) > free sugars (SACN).

### 4.3. Sources of Free Sugars

To our knowledge, there is no uniform definition of free sugars in the literature. The most common definition of free sugars used in studies [22,24,29] is based on the definition of WHO [3] and approaches vary in terms of the type of fruit products accepted as free sugars sources [20,22]. In the UK recently, Public Health England has expanded the broad definition set by SACN into a set of working principles for estimating the free sugar content of foods [30]. This free sugar definition of Public Health England [30] includes the sugar derived from vegetable puree on the advice that there was no scientific basis for treating processed fruit and vegetables differently [31]. In our study, the estimation of free sugars in foods was derived from fruit puree only. However, the same methodology can also be applied to the estimation of free sugars in foods derived from vegetable purees. Considering the relatively small impact of vegetable purees and fruit purees when estimated for a large population, researchers should decide themselves according to their research interest as to whether include or omit the estimation of free sugars coming from fruit and vegetable puree. Alternatively, researchers can make general estimations or disaggregate the complex foods which only belong to the food groups that are the main sources of fruit and vegetable puree for example commercial toddler foods and smoothies.

Users can also make rough estimation of free sugars from NMES content of foods [32] if resources are scarce or the aim is to do any practical interpretations based on NMES values published in previous studies and surveys.

### 4.4. Strength of the Method

The main strength of our method is the flexibility that it provides in estimating added sugars and free sugars in foods. For example, should a new definition propose the exclusion of fruit purees from a free sugar definition, this can be easily excluded from the summation of the free sugar components. Similarly, if an ingredient previously considered as free sugar is no longer considered one, the sugar composition of this ingredient can be altered in the Free Sugars Database. In addition, the method could help regulators make informed decisions on the implementation of different added sugars definitions by testing their effects at population level. Furthermore, our method provides the background estimations on added sugar and free sugars estimation in a transparent way due to disaggregation of complex foods and free sugars values. Another advantage of this method is that it enables food-specific estimations to be made. For example, if a user is interested in sugar intake derived from “dried fruit” they can derive this easily from the Nutrient Databank.

In the UK, the Soft Drinks Industry Levy [33] was introduced in 2016 as part of the Government’s childhood obesity strategy [9]. Our flexible method will enable the updating of free sugar values of foods consumed following 2016 to take account of any reformulation by manufacturers. Furthermore, our method is an ideal tool for manufacturers to estimate the added sugar and free sugar content of their products as they know the formulation.

Hidden (disguised) sources of added sugars (for example isoglucose) [34] are potentially important and a challenge for the management of added sugars intake for consumers [35]. Our method enables monitoring any potential of increasing hidden sources of sugars in food products as it includes the option to distinguish capture of sugar based sweeteners, such as fructose and syrups (sugar-other) from table and other sugars. However, if there is no particular interest to distinguish added sugars, users can estimate the added sugar value without making the distinction between table sugars and other sugars categories.

### 4.5. Limitation of the Method

Similar to other methods [19,20,22,28], the main limitation of this method is that the added sugar and free sugar content in food products could be under- or overestimated due to a degree of subjectivity introduced in some steps as there is no analytical way to differentiate added sugars and free sugars from the sugars naturally incorporated into the cellular structure of foods. In addition, the estimations made in this method relied on total sugars values, therefore the minimum requirement of this method is that the food composition database should provide quality data for total sugars values.

In added sugar and free sugar estimations, recipe calculation is an inevitable step, as in the method developed by Louie et al. [19]; however it can be challenging to develop a recipes database. Various countries or organizations use some form of datasets including standard recipes or the disaggregation of composite foods [36,37,38,39] which can also be used in a similar way equivalent to a recipe database. Technical database facilities such as the automated background calculation facilitate the recipe calculation process. Although some challenges are reported [23], linear programming can also be a promising approach in the future to streamline the recipe calculation process of the manufactured products. However, despite the challenges, our study highlights the additional benefits of maintaining an up-to-date recipes database alongside a country-specific food composition database.

## 5. Conclusions

This paper describes an improved method for the estimation of added sugar and free sugar content of foods with good accuracy and repeatability. The method allows for flexibility in measuring the values across multiple foods simultaneously and in a way which is applicable to variant definitions for sugars, and also provides the ability to make food-specific estimations. Due to its efficient and transparent structure, it could improve the capacity of food composition databases to continuously update the sugar values and enable the timely measurements in population surveillance studies. Our standardized approach can be beneficial to nutrition research and surveys to improve the monitoring of added sugar and free sugar intake as well as improving cross-study comparisons and can also assist food manufacturers with assessing the sugar content of formulations in accordance to different sugar recommendations.

## Figures and Tables

**Table 1:** Inter-rater repeatability test results; the comparison of mean values between two data analysts using paired *t*-test (*n* = 50).

	Data Analyst 1 (g/100 g)	Data Analyst 2 (g/100 g)	Difference of Means (g)	95% Confidence Interval		*p*
Sugar-table	15.98	15.88	0.10	−0.69,	0.89	0.80
Sugar-other	3.74	3.95	−0.21	−0.68,	0.25	0.36
Sugar-honey	0.43	0.47	−0.04	−0.24,	0.16	0.69
Sugar-fruit juice	0.76	0.74	0.02	−0.10,	0.15	0.69
Sugar-pureed fruit	0.77	0.73	0.04	−0.17,	0.24	0.73
Sugar-stewed fruit	0.98	1.10	−0.12	−0.27,	0.03	0.12
Sugar-dried fruit	0.72	0.70	0.02	−0.01,	0.04	0.21
**Added sugar and free sugar combinations**						
Added sugars ^1^	19.72	19.83	−0.11	−0.69,	0.47	0.70
Free sugars (WHO) ^2^	20.91	21.04	−0.13	−0.65,	0.40	0.63
Free sugars (SACN) ^3^	21.68	21.77	−0.09	−0.66,	0.48	0.75

^1^ Added sugars: sugar-table + sugar-other; ^2^ Free sugars (WHO): Added sugars + sugar-honey + sugar-fruit juice; ^3^ Free sugars (SACN): Free sugars (WHO) + sugar-pureed fruit. WHO: World Health Organization. SACN: Scientific Advisory Committee on Nutrition.

**Table 2:** Validation test results; the comparison of mean values between known formulations and their estimations using paired *t*-test (*n* = 50).

	Known Formulation * (g/100 g)	Estimation (g/100 g)	Difference of Means (g)	95% Confidence Interval		*p*
Sugar-table	15.98	15.84	0.14	−0.23,	0.50	0.45
Sugar-other	3.74	3.90	−0.16	−0.46,	0.13	0.27
Sugar-honey	0.43	0.33	0.10	−0.12,	0.32	0.37
Sugar-fruit juice	0.76	0.80	−0.04	−0.12,	0.05	0.37
Sugar-pureed fruit	0.77	0.72	0.04	−0.08,	0.17	0.49
Sugar-stewed fruit	0.98	1.12	−0.14	−0.29,	0.01	0.07
Sugar-dried fruit	0.72	0.69	0.02	−0.02,	0.07	0.32
**Added sugars and free sugars combinations**						
Added sugars ^1^	19.72	19.75	−0.03	−0.30,	0.24	0.85
Free sugars (WHO) ^2^	20.91	20.88	0.03	−0.14,	0.21	0.70
Free sugars (SACN) ^3^	21.68	21.60	0.08	−0.06,	0.21	0.25

* Known formulation also refers to values of recipes created by the data analyst-1 during inter-rater repeatability (see Table 1). ^1^ Added sugars: sugar-table + sugar-other; ^2^ Free sugars (WHO): Added sugars + sugar-honey + sugar-fruit juice; ^3^ Free sugars (SACN): Free sugars (WHO) + sugar-pureed fruit. WHO: World Health Organization. SACN: Scientific Advisory Committee on Nutrition.
